# Bee venom inhibits growth of human cervical tumors in mice

**DOI:** 10.18632/oncotarget.3110

**Published:** 2015-01-23

**Authors:** Hye Lim Lee, Sang Ho Park, Tae Myoung Kim, Yu Yeon Jung, Mi Hee Park, Sang Hyun Oh, Hye Seok Yun, Hyung Ok Jun, Hwan Soo Yoo, Sang-Bae Han, Ung Soo Lee, Joo Hee Yoon, Min Jong Song, Jin Tae Hong

**Affiliations:** ^1^ College of Pharmacy and Medical Research Center, Heungduk, Cheongju, Chungbuk, Republic of Korea; ^2^ Clinical Research Laboratory, Uijeonbu St. Mary's Hospital, The Catholic University of Korea College of Medicine, Uijeongbu, Republic of Korea; ^3^ College of Veterinary Medicine, Chungbuk National University, Heungduk, Cheongju, Chungbuk, Republic of Korea; ^4^ Department of Food Science & Technology, Korea National University of Transportation, Jeungpyeong, Republic of Korea; ^5^ Department of Obstetrics and Gynecology, St. Vincent's Hospital, College of Medicine, The Catholic University of Korea, Paldal-gu, Suwon, Gyeonggi-do, Republic of Korea; ^6^ Department of Obstetrics and Gynecology, Daejeon St. Mary's Hospital, College of Medicine, The Catholic University of Korea, Jung-gu, Daejeon, Republic of Korea

**Keywords:** bee venom, apoptosis, death receptors, NF-κB, cervical cancer

## Abstract

We studied whether bee venom (BV) inhibits cervical tumor growth through enhancement of death receptor (DR) expressions and inactivation of nuclear factor kappa B (NF-κB) in mice. *In vivo* study showed that BV (1 mg/kg) inhibited tumor growth. Similar inhibitory effects of BV on cancer growth in primary human cervical cancer cells were also found. BV (1–5 μg/ml) also inhibited the growth of cancer cells, Ca Ski and C33Aby the induction of apoptotic cell death in a dose dependent manner. Agreed with cancer cell growth inhibition, expression of death receptors; FAS, DR3 and DR6, and DR downstream pro-apoptotic proteins including caspase-3 and Bax was concomitantly increased, but the NF-κB activity and the expression of Bcl-2 were inhibited by treatment with BV in tumor mice, human cancer cell and human tumor samples as well as cultured cancer cells. In addition, deletion of FAS, DR3 and DR6 by small interfering RNA significantly reversed BV-induced cell growth inhibitory effects as well as NF-κB inactivation. These results suggest that BV inhibits cervical tumor growth through enhancement of FAS, DR3 and DR6 expression via inhibition of NF-κB pathway.

## INTRODUCTION

Bee venom (BV) therapy is the therapeutic application to the treatment of various diseases. BV has been used as an oriental medicine for the treatment of chronic inflammatory diseases, such as rheumatoid arthritis and pain [1–3]. Furthermore, it has been demonstrated that BV inhibits mammary carcinoma cell proliferation *in vitro* and tumor growth *in vivo*, such as prostate [4], ovarian [5], liver [6], bladder [7] and renal cancer cells [8] as well as leukemia cells [9]. However, there is a few available information on the effect of BV on human cervical cancer cells. Previous studies presented that simply inhibition of cancer cell growth and angiogenesis in culture cervical cancer cells [10, 11]. Therefore, our present study focused on xenograft model, clinical data using human sample as well as inhibition of human cancer cell growth. Cervical cancer is the third most common cancer among women and the second most frequent cause of cancer-related death worldwide [12]. Human papillomavirus (HPV) infection is a major cause of cervical cancer, however, HPV infection alone is not sufficient to cause cancer cells. Up-regulation of oncogenes and aberrant activation of related signals could be significant in cervical carcinogensis [13]. Thus, blocking agents these signals might be applicable for treatment of the development of cervical cancer.

Apoptotic cell death plays an important role in anti-cancer effects of chemotherapeutics. Stimulation of death receptor (DR) expression is implicated in the induction of apoptotic cell death in cancer cells and reduced chemoresistance of cancer cells. Eight members of the DR family have been characterized so far: tumor necrosis factor receptor 1 (TNFR1; also known as DR1, CD120a, p55 and p60), DR2 (also known as CD95, APO-1 and Fas), DR3 (also known as APO-3, LARD, TRAMP and WSL1), TNF-related apoptosis-inducing ligand receptor 1 (TRAILR1; also known as DR4 and APO-2), TRAILR2 (also known as DR5, KILLER and TRICK2), DR6, ectodysplasin A receptor (EDAR) and nerve growth factor receptor (NGFR) [14, 15]. When DRs bind to their ligands, the death domains recruit the intracellular adaptor protein FADD (Fas-associated death domain protein) which results in the activation of caspases, including caspases-3, -8 and -9, as well as increase of Bax and decrease of Bcl-2 [16, 17]. These extrisinc apoptotic signals are signigficant in chemotherapeutic-induced selective apoptosis of cervical cancer [18, 19]. Moreover, much higher expression of DRs in cervical cancer cell lines and human cervical tumor tissues compared to that in normal cervical tissues have been reported [20]. Therefore, stimulation of DR expression could be significant for chemotherapy as well as reduced chemoresistance of cervical cancer cells.

The nuclear factor kappa B (NF-κB) family plays an important role in several human cancer cell growth [21, 22]. NF-κB activation participates at multiple steps in tumor growth and its suppression leads to the suppression of tumor development. NF-κB mediates the expression of genes that are involved in tumor promotion, angiogenesis, and metastasis [23, 24]. Several tumor types including cervical cancer show a persistent constitutive activation of NF-κB, and NF-κB activation has been shown to induce resistance to various chemotherapeutic agents [25, 26]. Cervical cancer also regulates cell growth through the activation of NF-κB [27] and higher NF-κB activity contributes to chemotherapeutic resistance of cervical cancer cells [25, 28, 29]. NF-κB has been also significantly associated with the up-regulation of pro-apoptotic DRs such as Fas, FasL and DR3-DR6 in several cancer [30, 31]. We previously found that BV inhibited prostate and ovarian cancer cell growth by inhibiting NF-κB, but up regulation of DR pathways [4, 5]. In the present study, we investigated whether BV has also anti-cancer effect in cervical cancer cells through modification of NF-κB and DR pathways in *in vivo* xenograft mice model, human tumor tissues, human primary cervical cancer cells as well as cervical cancer cell lines.

## RESULTS

### BV inhibited tumor growth *in vivo* xenograft

**Figure 1:**
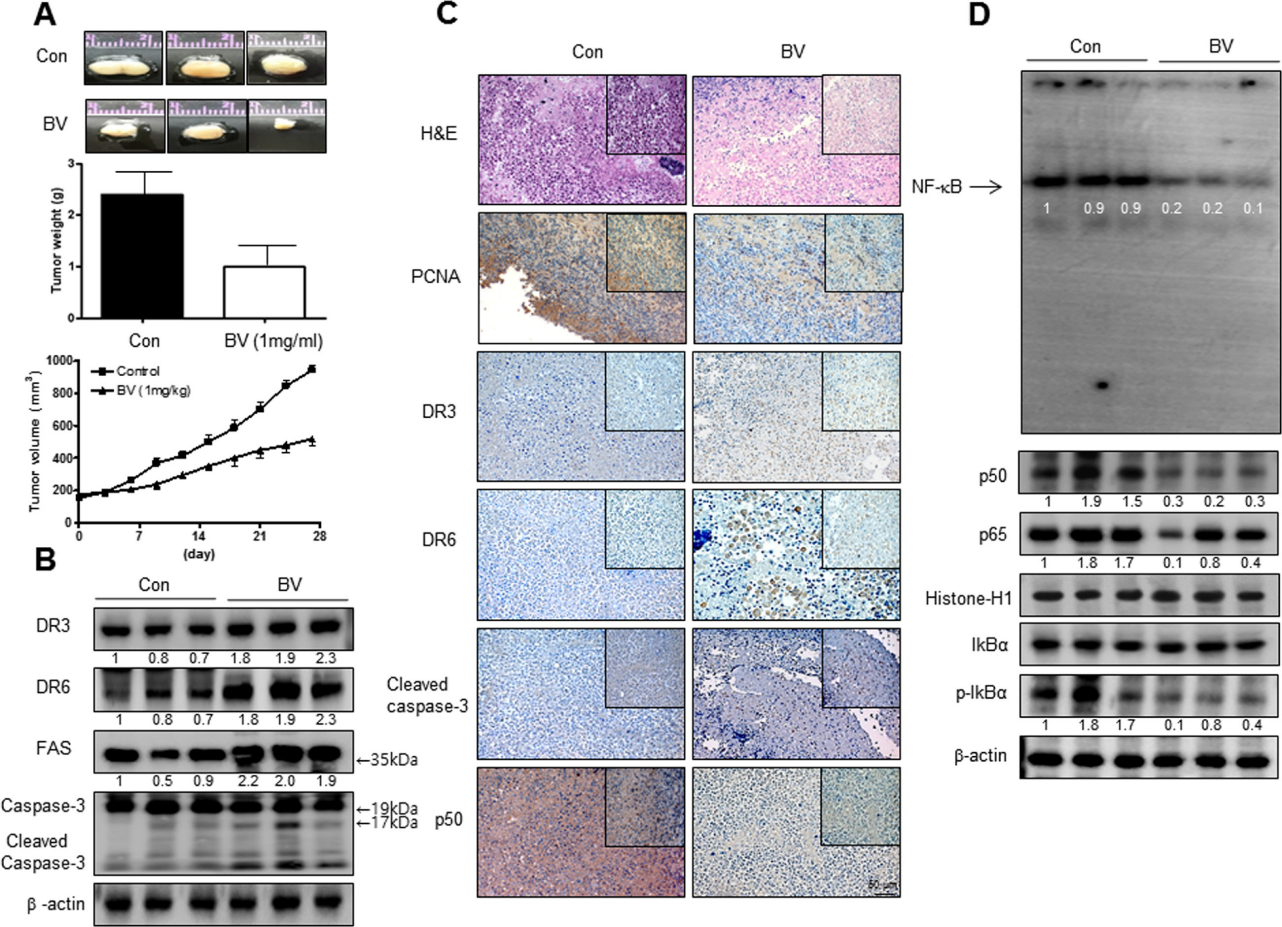
Anti-tumor activity of BV in cervical cancer xenograft Growth inhibition (as assessed by tumor volume) of subcutaneously transplanted CA Ski xenografts mice treated with BV (1 mg/kg/ two times a week) for 4weeks. Xenografted mice (*n* = 10) were administrated intraperitoneally with saline (1 ml/kg) or BV (1 mg/kg). Tumor burden was measured once per week using a caliper, and calculated volume length (mm) × width (mm) × height (mm)/2. Tumor weight and volume are presented as means ± S.D. **(A)**. The expression of apoptotic proteins was detected by western blotting using specific antibodies; DR3, DR6, FAS, cleaved caspase-3 **(B)**. β-actin protein was used an internal control. Immunohistochemistry was used to determine expression levels of H&E, PCNA, DR3, DR6, p50 in nude mice xenograft tissues by the different treatments as described in the Materials and Methods Section **(C)**. NF-κB activity in tumor tissues **(D)**. All values represent mean ± SD from five animal tumor sections. *P < 0.05 indicates significantly different from the control group.

To elucidate the anti-tumor effect of BV *in vivo*, the tumor growth on cervical cancer cell xenograft bearing nude mice following BV (1 mg/kg) treatments was investigated. Since our previous studies in other cancer cells, 3 mg/kg was effective [4], thus, we used 1 mg/kg BV in cervical cancer. In Ca Ski xenograft studies, BV was administrated intraperitoneally two times per week for 4 weeks to mice which have tumors ranging from 100 to 300 mm^3^. Tumor volume was measured weekly, and all mice were killed at the end of experiment when tumors were dissected and weighted. The inhibitory effect of BV on growth of cervical tumor was significant in xenograft model mice (Figure 1A upper panel). Tumor weight and volume were significantly smaller in 1 mg/kg BV-treated compared with those saline-treated Ca Ski bearing mice (Figure 1A middle and lower panel). Expression of FAS, DR3 and DR6 and DR downstream pro-apoptotic proteins including cleavaged caspase-3, -8 and -9 was concomitantly increased (Figure 1B), but the NF-κB activity and expression of pI03BAB and nucleus p50 and p65 were inhibited in tumor tissues by treatment with BV (Figure 1C).

### Expression of DR and cleaved caspases, as well as NF-κB activity in human cervical tumor tissues

**Figure 2:**
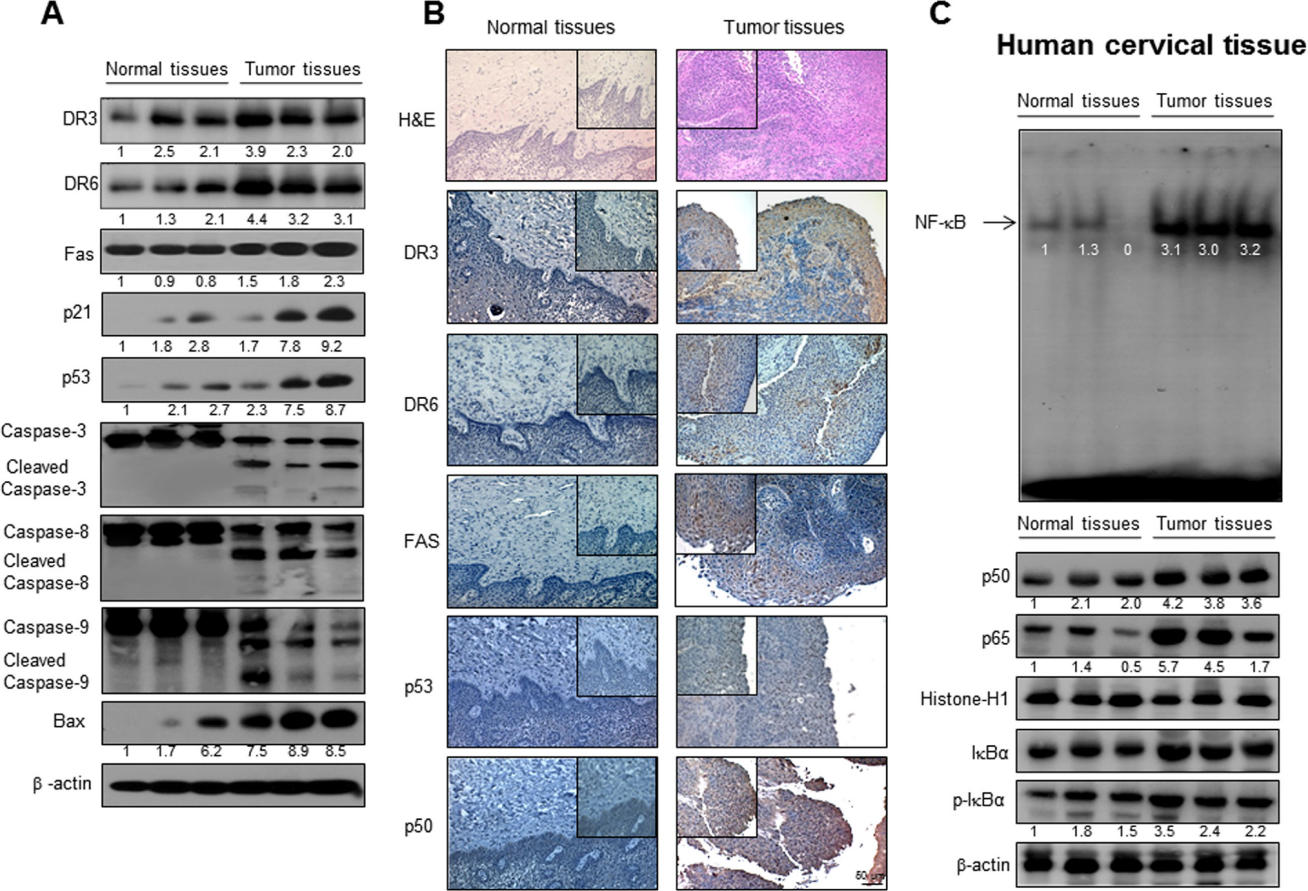
Expression of apoptosis regulatory proteins and DRs, and NF-κB activity in human cervical tissues The expression of apoptotic proteins was detected by Western blotting using specific antibodies; DR3, DR6, FAS, p53, Bcl-2, cleaved caspase-3, cleaved caspase-8, cleaved caspase-9, Bax and β-actin in the cervical cancer tissues and cervical normal tissues **(A)**. β-actin protein was used an internal control. Immunohistochemistry analysis of FAS, DR3 and DR6 confirmed that the intensities of nuclear staining for DR3, DR6 and FAS were decreased in the cervical tissues (containing tumor tissues) of human **(B)**. Activation of NF-κB in the cervical cancer tissues and cervical normal tissues **(C)**. Nuclear extract from cervical cancer tissues was incubated in binding interaction of 32P-end-labeled oligonucleotide containing the κB sequence. The cervical tissues was incubated and were lysed, cytosolic proteins were used to determine the expression of IκB, p-IκB (internal control) and nuclear proteins were used to determine the expression of p50, p65 and Histone-H1(internal control) in cervical tumor tissues.

To examine the relationship between human tumor growth and DR expression and NF-κB activity, we compared expression of FAS, DR3, DR6 and expression of p21, p53, cleavaged 3, 8 and 9 as well as NF-κB activity between normal human cervical tissues and human tumor tissues. We found that much higher elevated expression of FAS, DR3, DR6, p21 and p53 as well as cleavaged caspase -3, -8 and -9 in cervical tumor tissues compared to those in normal tissues detected either by Western blotting and immunohistochemistry (Figure 2A and 2B). We also found the NF-κB activity, pIκB expression and nucleus expression of p50 and p65 was much higher in human tumor tissues compared to that in normal cervical tissues (Figure 2D).

### Effect of BV on cultured primary human cervical cancer cell growth, NF-κB activity and DR expression

**Figure 3:**
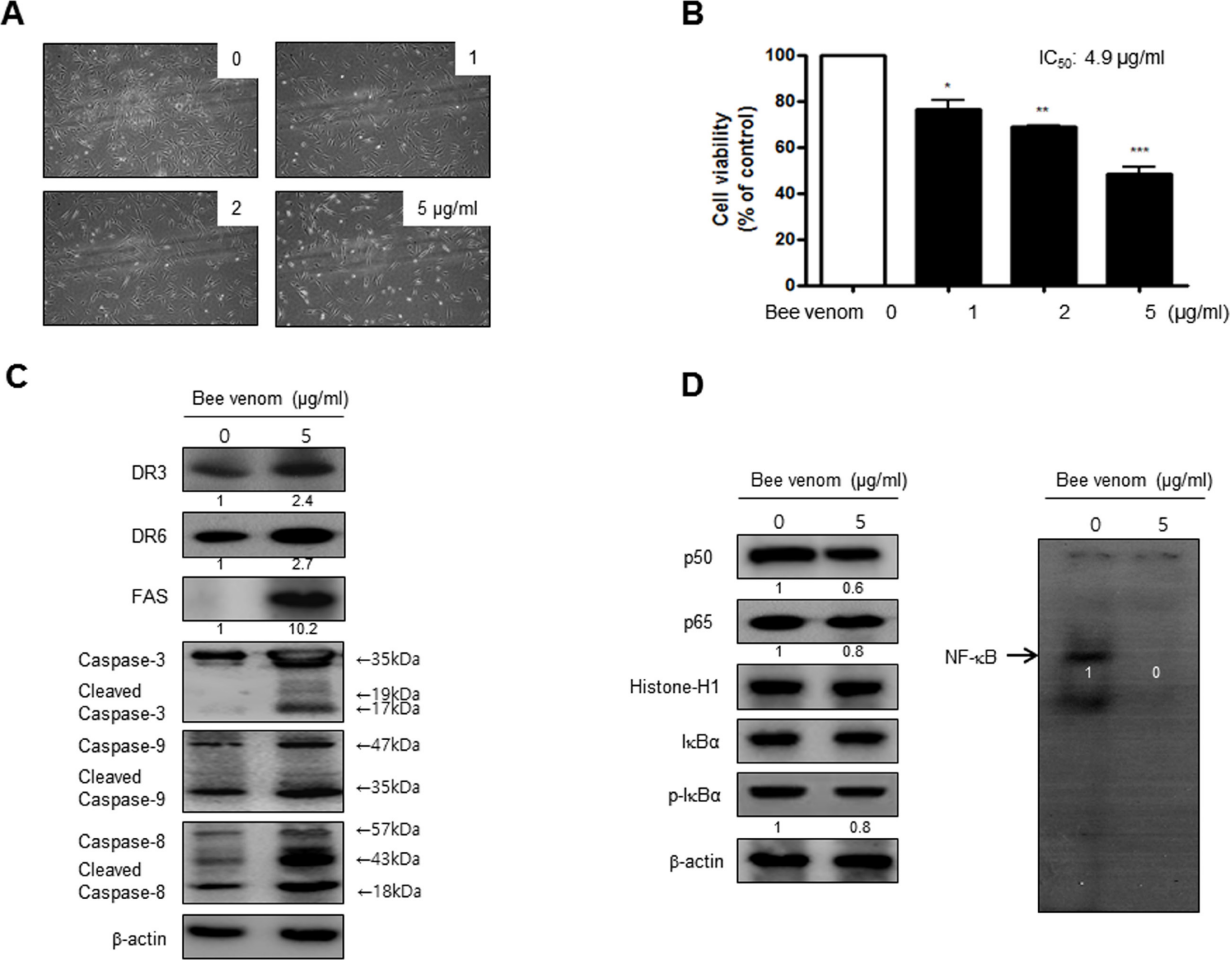
Effect of BV on cell viability and morphological changes of human primary cervical cancer cells Morphologic observation with the treatment of BV. Human primary cervical cancer cell changes were observed under phase contrast microscope **(A)**. The data **(B)** are expressed as the mean ± S.D. of three experiments. *(*P* < 0.05) indicates statistically concentration-dependent effect of BV **(A)** on the MTT viability assay in human primary cervical cancer cell. Expression of apoptosis regulatory proteins related extrinsic pathway was determined using Western blot analysis **(C)**, NF-κB activity and expression of related proteins determined by EMSA or Western blot **(D)** were determined as similar to the cancer cell lines. Each band is representative for three experiments.

To investigate the inhibitory effect of BV (5 μg/ml) on primary human cervical cancer cell growth, we cultured primary human cervical cancer cells with or without BV. Similar to cancer cell lines, we found that BV clearly inhibited growth of primary cultured human cervical cancer cells with IC_50_ value of 4.9 μg/ml (Figure 3A and 3B). Western blot analysis showed that BV treatment clearly increased FAS, DR3 and DR6 expression as well as cleavaged caspase-3, -8 and -9 (Figure 3C). NF-κB was also constitutively activated in primary human cervical cancer cells. However, inhibition of NF-κB activity as well as phosphorylation of IκB was also found in cultured primary human cervical cancer cells by the treatment of BV (Figure 3D).

### Effect of BV on cervical cancer cell growth

**Figure 4:**
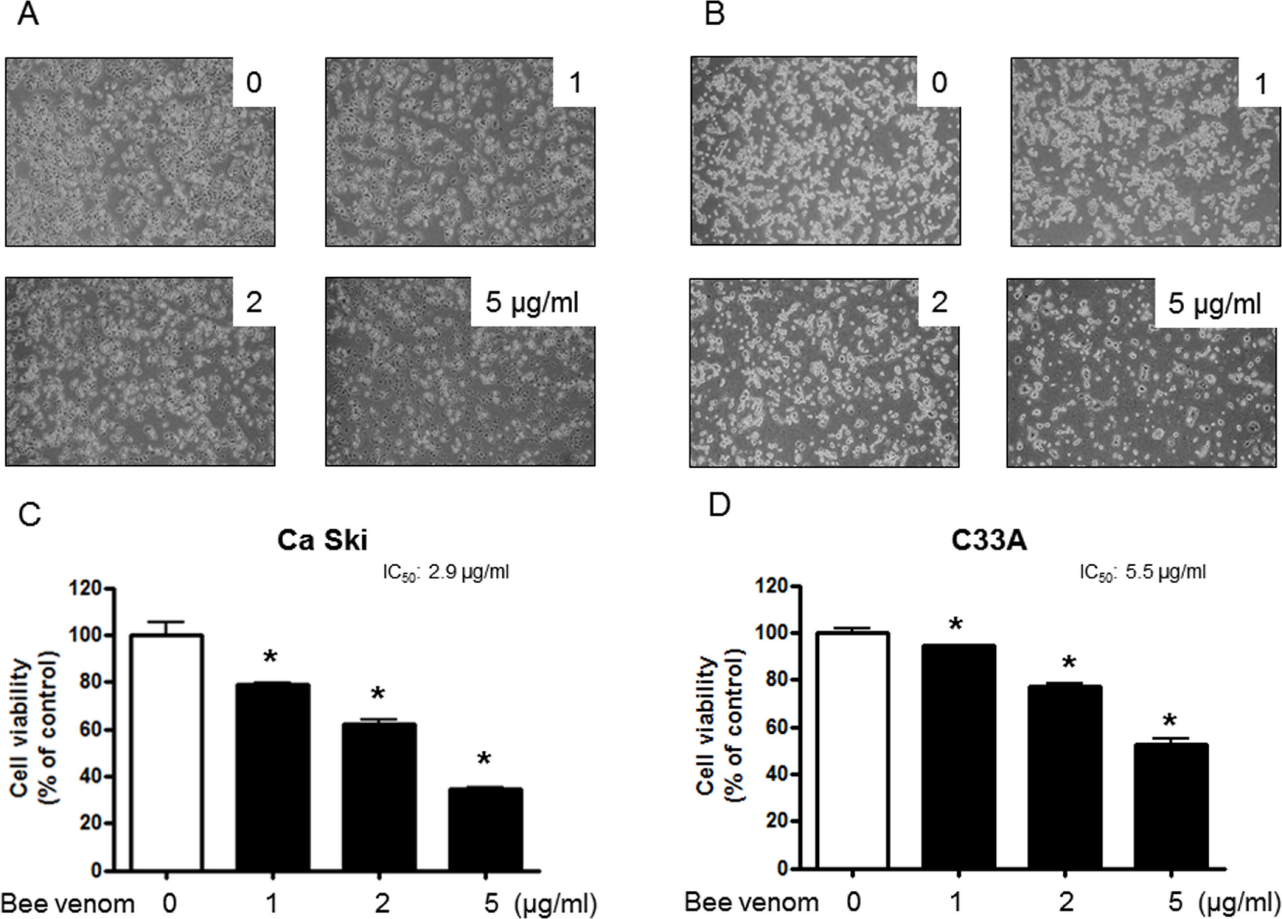
Effect of BV on cell viability and morphological changes of cervical cancer cells Concentration-dependent effect of BV on the MTT viability assay in Ca Ski and C33A after 24 hr. Morphologic observation with the treatment of BV. Ca Ski and C33A cells morphological changes were observed under phase contrast microscope **(A** and **B)**, respectively). The data were expressed as the mean ± S.D. of three experiments. *(*P* < 0.05) indicates statistically significant differences from the control group.

To assess the inhibitory effect of BV on cell growth of cervical cancer cells Ca Ski and C33A cells, we analyzed cell viability by MTT assay. The cells were treated with several concentrations of BV (1, 2, and 5 μg/ml) for 24 hr. As shown in Figure 1, BV inhibited cell growth of cervical cancer cells in a concentration-dependent manner. BV (0–5 μg/ml) inhibited growth of human cervical cancer cells; Cs Ski (Figure 4C) and C33A (Figure 4D) with IC_50_ values of 2.9 and 5.5 μg/ml respectively. Morphologic observation indicated that the cells were gradually reduced in size and showed a small round single cell shape by the treatment of BV in Ca Ski cells (Figure 4A) and C33A cells (Figure 4B). We also found that BV inhibited other cancer cell growth such as lung, ovarian and colon cancer cells with different IC_50_ values (Supplementary Figure 1).

### Effect of BV on apoptotic cell death

**Figure 5:**
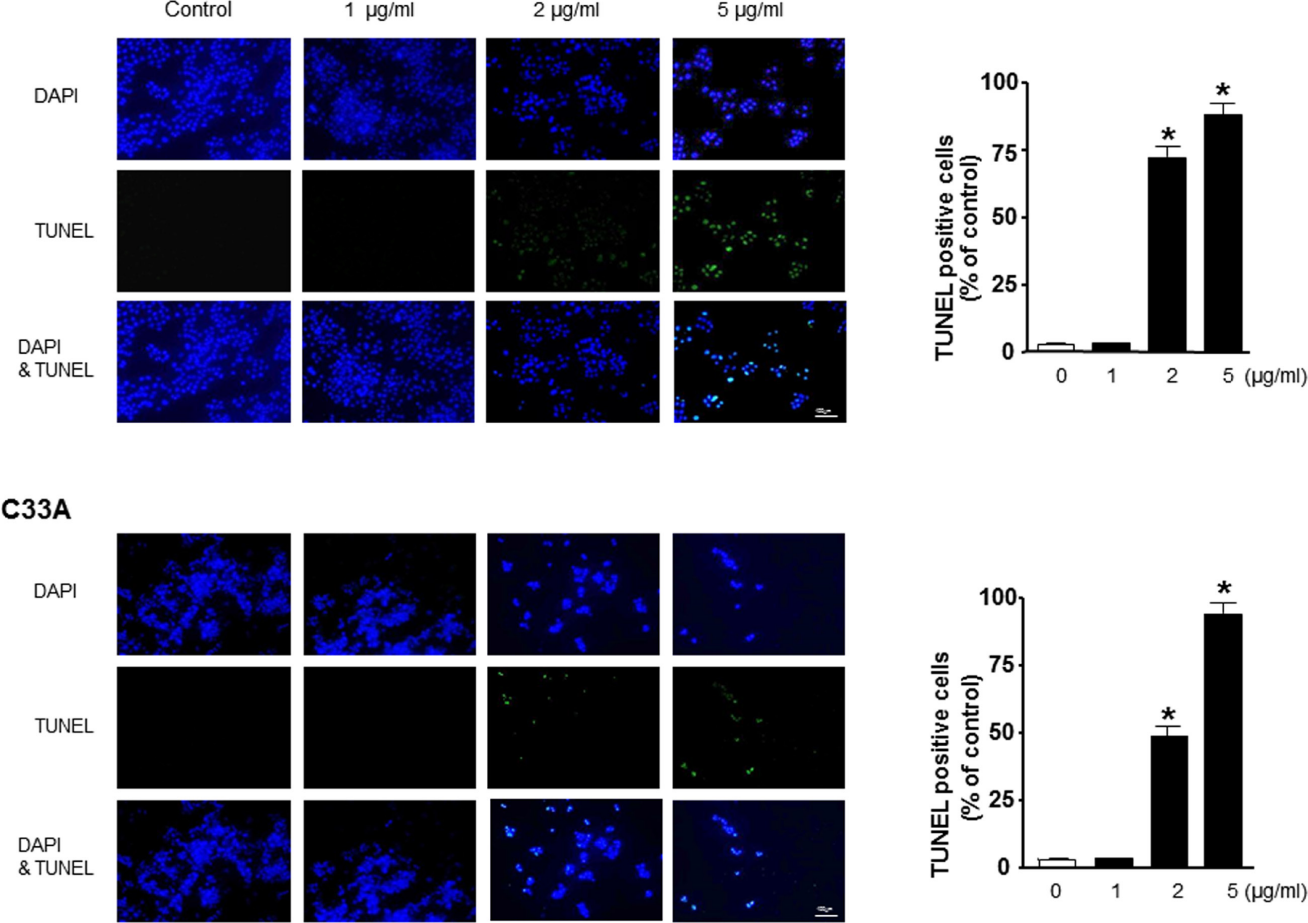
Effect of BV on apoptotic cell death The cervical cancer cells were treated with BV for 24 hr, and then labeled with DAPI and TUNEL solution. Total number of cells in a given area was determined by using DAPI nuclear staining (fluorescent microscope). The green color in the fixed cells marks TUNEL-labeled cells. The apoptotic index was determined as the DAPI-stained TUNEL-positive cell number/total DAPI stained cell number (magnification, 200 ×). Values were means ± S.D. of three experiments. *(*P* < 0.05) indicates statistically significant differences from the control cells.

We performed DAPI staining followed by TUNEL staining assays, and then the double labeled cells were analyzed by fluorescence microscope to determine the inhibition of cell growth by BV was due to the induction of apoptotic cell death. Reversely consistant with cell growth inhibitory effects, apoptotic cell death was significantly increased in BV treated Ca Ski and C33A cervical cancer cells, respectively (Figure 5). The number of apoptotic cells (DAPI-positive TUNEL-stained cells) in CA Ski and C33A human cervical cancer cell cultures was increased to about 87% and 90% of cells, respectively at a concentration of 5 μg/ml (Figure 5B).

### Effect of BV on expression of DR and apoptotic regulatory proteins

**Figure 6:**
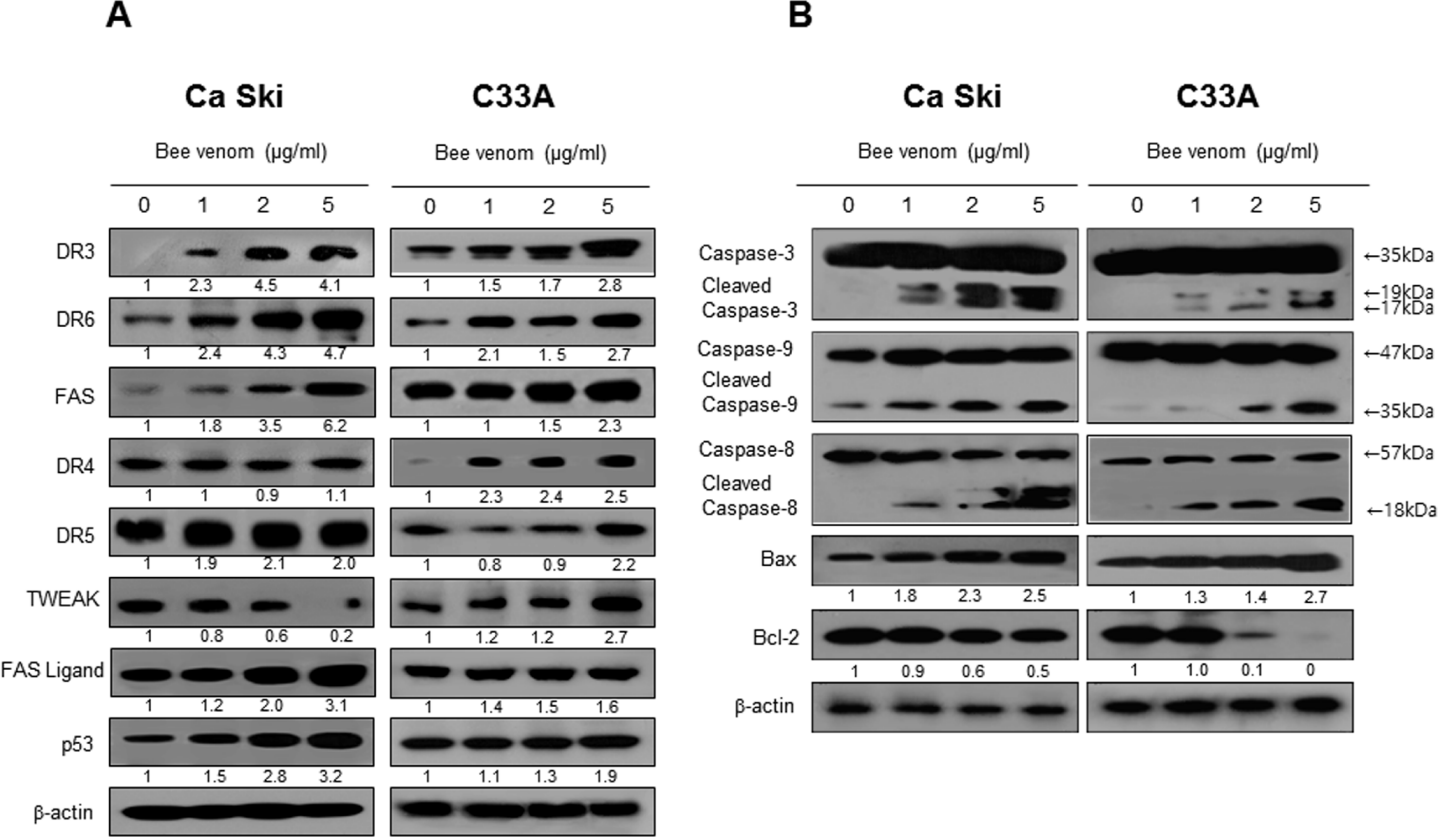
Effect of BV on the expression of apoptosis regulatory proteins Expression of apoptosis regulatory proteins related exntrinsic pathway was determined using Western blot analysis with the antibodies against DR3, DR6, FAS, TWEAK, TRAIL, FAS ligand, p53, caspase-3, caspase-8, caspase-9, Bax, bcl-2 and β-actin. β-actin protein was used an internal control. Each band is representative for three experiments.

Apoptotic cell death can be induced by increase expression of DRs. Therefore, to investigate expression of DRs in cervical cancer cells undergoing apoptotic cell death, we performed Western blot analysis. Western blot analysis showed that BV treatment clearly increased DR3 and DR6 expression in a concentration dependent manner in both cells, and expression of FAS in Ca Ski and DR4 in C33A cancer cells was increased by BV (Figure 6A).

### Effect of BV on expression of apoptotic regulatory proteins

To figure out the relationship between the induction of apoptotic cell death, and the expression of their regulatory proteins by BV, expression of apoptotic cell death related proteins was investigated by Western blots. The expression of anti-apoptotic protein Bcl-2 was decreased; however, the expression of pro-apoptotic proteins, Bax, cleaved form of caspases-3, -8, and -9 was increased by treatment of BV in a concentration dependent manner (Figure 6B).

### Effect of BV on NF-κB activation

**Figure 7:**
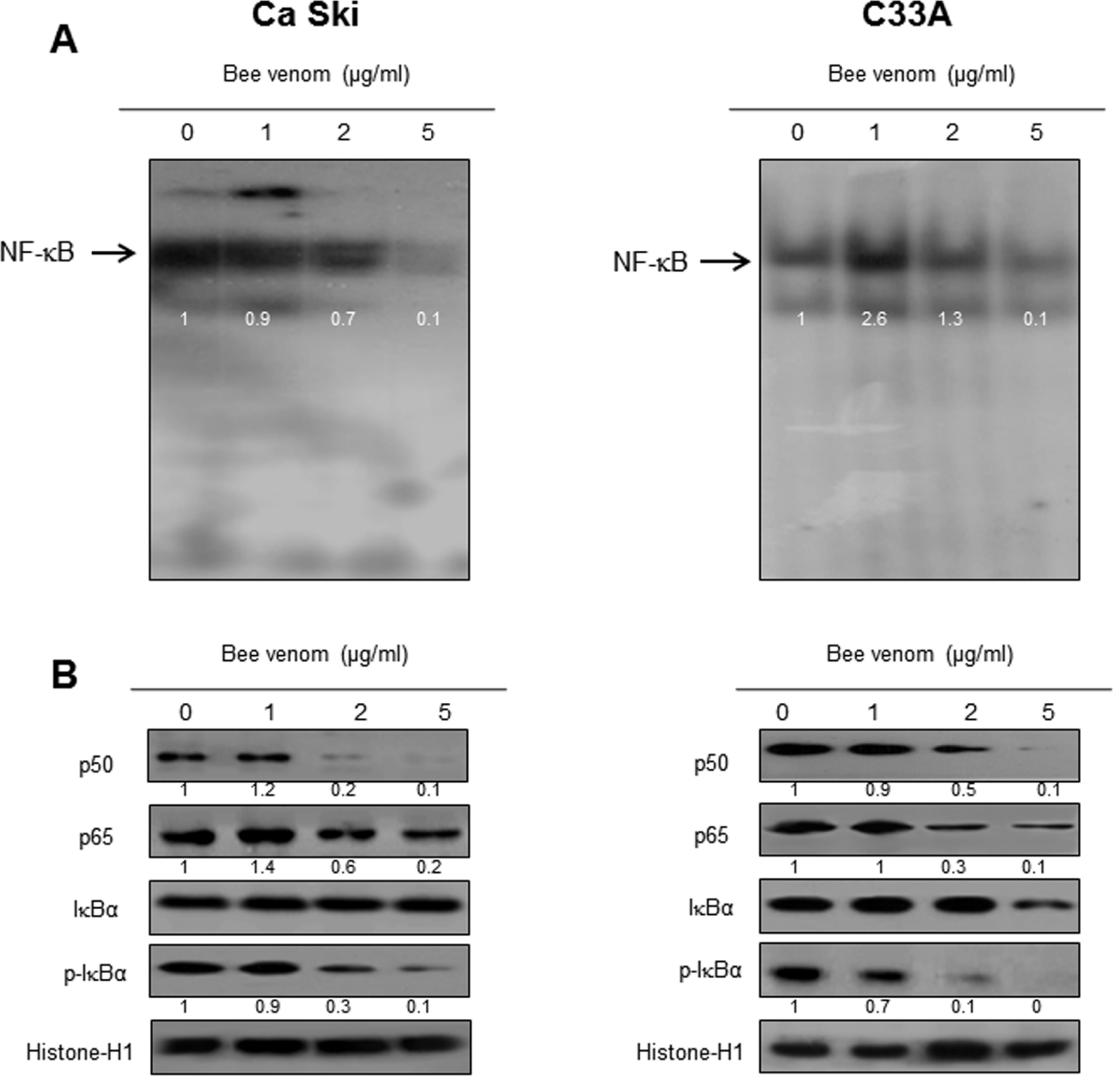
Effect of BV on NF-κB activation Nuclear extract from cervical cancer cells treated with BV (1, 2, and 5 μg/ml) for 2 hr was incubated in binding interaction of 32P-end-labeled oligonucleotide containing the NF-κB sequence. The present EMSA results are representatives of three experiments **(A)**. The cells treated with BV (1, 2, and 5 μg/ml) for 2 hr was incubated and were lysed, cytosolic proteins were used to determine the expression of IκB, p-IκB (internal control) and nuclear proteins were used to determine the expression of p50, p65 and Histone-H1(internal control) in cervical cancer cells **(B)**. Each band is representative for three experiments.

NF-κB is significant in cervical cancer cell growth. To investigate whether BV inactivates NF-κB, we did EMSA for detecting DNA binding activity of NF-κB. We found that BV untreated cervical cancer cells showed highly constituted activation of NF-κB in both cervical cancer cells. However, the treatment of BV dose dependently inhibited DNA binding activity of NF-κB (Figure 7A). Agreed with the inhibition of NF-κB, cytosolic phosphorylation of IκB as well as the nucleus expression of p50 and p65 were inhibited by BV treatment in both cervical cancer cells (Figure 7B).

### Reversed effect of DR siRNAs on BV-induced cell growth inhibition and NF-κB inactivation

**Figure 8:**
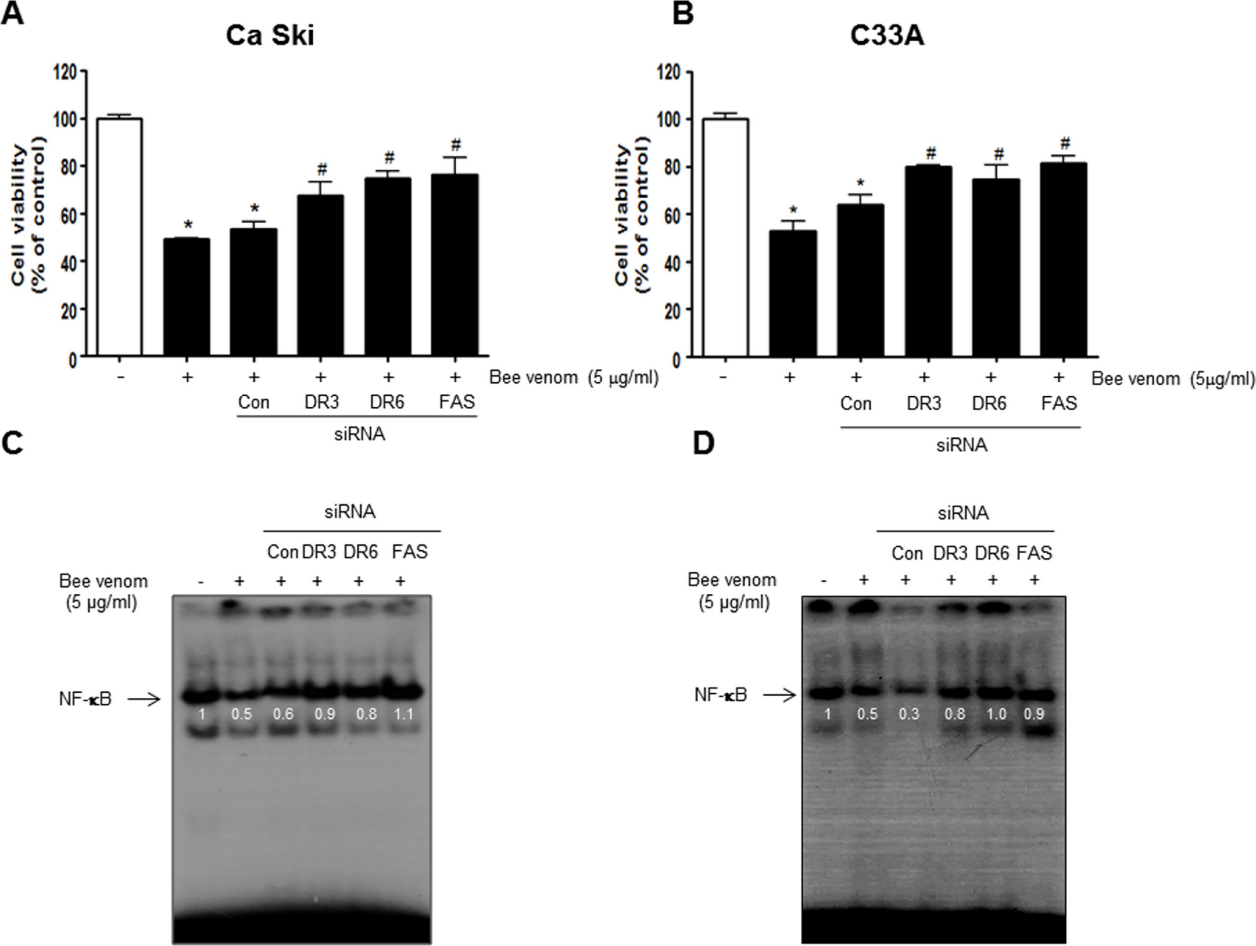
Effect of siRNA of DR3, DR6 and FAS on the BV induced cancer cell growth inhibition, and NF-κB activity in cervical cancer cells The cervical cancer cells were transfected with the DR3, DR6 and FAS siRNA (10 nM) for 24 hr, the cells were then and treated with BV (5 μg/ml) for 24 hr. After treatment, cell viability was measured by MTT assay **(A, B)** and NF-κB activity **(C, D)** determined as described above. Cell growths are means ± S.D. of three experiments. *(*P* < 0.05) indicates statistically significant differences from control group. #(*P* < 0.05) indicates statically significant differences from BV treated group.

To determine the relationship between DR expression and cervical cancer cell growth inhibitory effect of BV, we transfected Ca Ski and C33A cells with DR siRNA using a transfection agent. The cells were transfected with 100 nM siRNA of DRs for 24 hr, and then treated with BV(5 μg/ml) for 24 hr. Knock down of DRs(FAS, DR3, DR6) almost completely reversed the cell growth inhibitory effect of BV in Ca Ski (Figure 8A) and C33A (Figure 8B). We also evaluated NF-κB activity. Inhibition of NF-κB by BV was abolished by transfection with FAS, DR3, DR6 siRNA both in Ca Ski (Figure 8C) and C33A cells (Figure 8D).

## DISCUSSION

In the present study, we found that BV clearly inhibited growth of primary human cervical cancer cells and tumor growth in xenograft female BALB/c nude mice model accompanied with increased DR3 and DR6 expression and NF-κB inactivation. Moreover, we found that significant higher expression of DR3, DR6 and p50 as well as NF-κB activity in human cervical tumor tissues. We also found that BV inhibited cell growth of human cervical cancer cells; Ca Ski and C33A through induction of apoptotic cell death via increase of DR3 and DR6 expression. In addition, BV inhibited other cancer cell growth with different efficacy. Thus, the present data indicates that BV could be effective for treatment of cervical cancer through up-regulation of FAS, DR3 and DR6, but inactivation of NF-κB. It has been demonstrated that BV inhibits cell growth in several types of cancer cells including cancer, prostate [4], ovarian [5], liver [6] and bladder cancers [7]. In addition to the inhibitory effect on these cancer cells, our data indicate that BV has also anti-cancer effect in cervical cancer cells. The effectiveness of BV in cervical cancer cells (dosage of anti-cancer effect) are similar to the dosage in other cancer cells such as colon, prostate [4] and ovarian [5]. Even though, the cell growth inhibitory effect of BV in p53 positive Ca Ski cells was much greater than p53 negative C33A cells, we did not see any difference in the dosage to inhibit primary human cervical cancer cell growth inhibition. Thus, the p53 dependent intrinsic apoptosis pathway may not be significant in BV-induced cervical cancer cell growth inhibition. It is known that about 50% of human cancer possess a p53 genomic mutation that may enable to escape from death induced by conventional therapeutic agents [32]. In the case of cervical cancer, it appears to be significant because E6 HPV protein leads to down regulation of p53, thereby the possibility that give some cervical carcinomas escape from apoptosis via intrinsic pathway. Thus, development of novel agents that exploit the extrinsic apoptotic pathway become to be significant.

Several studies have demonstrated that natural compounds-induced cell growth inhibition in cancer cells could be related with increase of DR pathway, an important extrinsic apoptotic pathway. Indomethacin and sulindac sulfide, one of the major metabolites of sulindac, activate caspase 8 and induce apoptosis by a fas-associating protein with death domain (FADD)-dependent mechanism in Jurkat T cells [16]. Sulindac sulfide is also believed to mediate its antitumorigenic effects by inducing apoptosis through up-regulated DR5 and activated the caspase 8 in colon and prostate cancer cell lines [17]. Our previous study also showed that BV and Snake venom toxin induced apoptotic cell death of colon, prostate, and ovarian cancer cells through enhancement of DRs (DR3, DR4, DR5 and DR6) expression. Our present results showed that expression of DR proteins such as DR3 and DR6 in Ca Ski and C33A cervical cancer cell were increased. However, treatment of DR3 and DR6 siRNA in Ca Ski and C33A reversed BV-induced cervical cancer cell growth inhibition. We also found that DR3 and DR6 expression was significantly higher in the BV treated cultured human cervical cancer cells as well as cervical tumor tissues and xenograft tumor tissues. Selective triggering of DR expression is implicated in the induction of cancer cell death and the expression of DR is varying depending on cell types and stimuli conditions. These data indicated that higher DR3 and DR6 expression could be significant for anti-cancer effect of BV.

It is well known that NF-κB is constitutively activated in cervical cancers [33]. Agreement with this notion, our data demonstrated that activation of NF-κB was elevated in human cervical tumor tissue, primary human cervical cancer as well as cervical cancer cell lines. However, treatment of BV inhibits cervical cancer cell growth accompanied with the inhibition of DNA binding activity of NF-κB. The decrease of NF-κB DNA binding activity was associated with the inhibitory effect of BV on the IκB phosphorylation and nuclear translocation of p50 and p65 in Ca Ski and C33A cells. These inhibitory effect of BV on NF-κB were agreed with other our studies [2, 3]. In these studies, we showed that BV interacts with p50 submit of NF-κB, thus decreased NF-κB DNA binding activity. NF-κB activation and p50 expression were also lowered in BV treated primary human cervical cancer cells and xenografted tumor tissues treated by BV. Thus, inactivation of NF-κB could be significant in BV-induced cervical cancer cell growth inhibition. Death signaling may be antagonized by anti-apoptotic modulator proteins of the Bcl-2 family [25]. BV also repressed the expression of anti-apoptotic proteins (Bcl-2), whereas it increased the expression of pro-apoptotic proteins (Bax, cleaved caspase-3, and cleaved caspase-9) which are regulated by NF-κB. Thus, BV may induce an alteration of apoptosis and anti-apoptosis regulatory protein expression that provide the favorable circumstance of the cancer cells to go to a death status by down regulation of NF-κB. It has been demonstrated that DR up-regulation and NF-κB inactivation reciprocally associated in cancer cell growth inhibition by Fisetin in human pancreatic cancer cells [30] as well as avidin treated human metastatic SW620 cells [31]. Our previous studies found that BV increased DRs expression, but inhibited NF-κB activity in prostate and ovarian cancer cells [4, 5]. We also founded that treatment of BV with DR3 and DR6 siRNA reversed inactivation of NF-κB and cell growth inhibitory effects of BV in cervical cancer cell lines. Other studies also found that turmeric inhibits human colon adenocarcinoma cells through increased expression of DRs (DR4 and DR5) with suppression of NF-κB activation [26]. Thus, reduced NF-κB activity could be associated with the inhibition of cervical cancer cell growth through up-regulation of DR3 and DR6.

It is reported that main side effects of BV has local and systemic allergic reactions. In recent years, it is also reported that BV cause uterine contractions as side effect [34, 35]. The LD_50_ value for BV in mice is 7.4 mg/kg [36]. We reported that BV has safe therapeutic index (1.2–2.47) [4]. BV (1 mg/kg) treatment did not cause any serious health problems such as eruption, swelling body weight loss or death. Eventhough these side effects were reported in the specific cases and higher dose treated, it could be safe by cautious practice. In conclusion, our results that natural toxin BV could be useful as an anti-cancer agent through activation of extrinsic apoptosis pathway by overexpression of FAS, DR3 and DR6, and by inactivation of NF-κB for treatment of cervical cancer cells.

## MATERIALS AND METHODS

### Materials

BV was purchased from You-Miel BV Ltd. (Hwasoon, Jeonnam, Korea). The composition of the BV was as follows: 45–50% melittin, 2.5–3% mast cell degranulating peptide, 12% phospholipase A2, 1% lysophospholipase A, 1–1.5% histidine, 4–5% 6-pentyl a-pyrone lipids, 0.5% secarpin, 0.1% tertiapin, 0.1% procamine, 1.5–2% hyaluronidase, 2–3% amine, 4–5% carbohydrate, and 19–27% of others, including protease inhibitor, glucosidase, invertase, acid phosphomonoesterase, dopamine, norepinephrine, and unknown amino acids, with 99.5% purity.

### Animal xenografts

To conduct *in vivo* studies, female BALB/c nude mice (aged 6–7 weeks, weighing 20–25 g) were used. Nude mice were housed under specific pathogen free conditions according to the guidelines of the Animal Care Committee at the Chungbuk National University (CBNU-278-11-01). On day 0, Ca Ski cells in PBS (2 × 10^7^ tumor cells/ 0.1 ml PBS/ani-mals) were injected subcutaneously into nude mice. BV (1 mg/kg) was administrated intraperitoneally twice per week for 4 weeks to mice which have tumors ranging from 100 to 300 mm3. Tumor volumes were estimated by the formula: length (mm) × width (mm) × height (mm)/2 at the end of experiment.

### Histopathology and immunohistochemistry

The cervical human tissues and animal tissues were fixed in 4% paraformaldehyde and cut into 30 μm sections using a freezing microtome (Thermo Scientific, Germany). The sections were stained with hematoxylin and eosin (H&E) for pathological examination. For immunohistological staining, tumor sections were incubated with primary antibody against FAS, DR3, DR6 and p50 (1:500, Abcam, Cambridge, UK). After rinse in phosphate buffered saline (PBS), the sections were subject to incubation in biotinylated secondary antibody. The tissue was incubated for 1 hr in an avidin-peroxidase complex (ABC, Vector Laboratories, Inc., Burlingame, CA). After washing in PBS, the immunocomplex was visualized using 3, 3-diaminobenzidine solution (2 mg/10 ml) containing 0.08% hydrogen peroxide in PBS. Sections were dehydrated in a series of graded alcohols, cleared in xylene and coverslipped using Permount (Fisher Scientific, Suwanee, GA).

### Primary human cervical cancer cell culture

After surgery, fresh tissue collected in cold phosphate buffered saline (PBS) and washed with PBS. Several small pieces of minced tumor tissue were incubated in phenol-red free DMEM/F12 (20 ml) containing type I collagenase and DNase I for 3~5 hr at 37°C with shaking. It was filtrated through a 100 μm nylon cell strainer (BD) for 2 times and 70 μm nylon cell strainer for 1 times. After filtration, cells remaining in the filtrate were collected by centrifugation at 1500 rpm for 5 min and washed with PBS. Primary cells were resuspended in phenol-red free DMEM/F12, and plated into 100 mm^2^ dishes.

From July 2012 to July 2013, seven patients affected with invasive cervical cancer (squamous cell carcinoma) and one healthy control donor, were enrolled prospectively from the department of obstetrics and gynecology of the Daejeon St. Mary's Hospital, Catholic University of Korea, using a research protocol approved by our Institutional review board (DC12TISI0044). Informed consent was provided according to the declaration of Helsinki. All patients underwent standardized treatment for their disease, which included radical hysterectomy, concurrent chemoradiation and chemotherapy. Samples were collected at the time of diagnosis. In this study, we examined 7 patients presenting with the international federation of gynecology and obstetrics (FIGO) stages IIA–IIB cervical cancers.

### Cervical cancer cell culture

Ca Ski and C33A human cervical cancer cells were obtained from the American Type Culture Collection (Cryosite, Lane Cove NSW, Australia). Cells were grown in DMEM (Gibco, Life Technologies, Grand Island, NY) with 10% fetal bovine serum, 100 U/ml penicillin, and 100 μg/ml streptomycin at 37°C in 5% CO_2_ humidified air. Primary human cervical cancer cells obtained from patients cervical tumor were grown in DMEM/F12 (Gibco, Life Technologies, Grand Island, NY) with 10% fetal bovine serum, 100 U/ml penicillin, and 100 μg/ml streptomycin at 37°C in 5% CO_2_ humidified air.

### Measurement of cell viability

Cervical cancer cells, Ca Ski and C33A cells were plated in 96-well plates, and subsequently treated with BV (0~5 μg/mL) for 24 hr. After treatment, cell viability was measured by MTT [3-(4,5-Dimethylthiazol-2-yl)-2,5-Diphenyltetrazolium Bromide] assay (Sigma Aldrich, St. Louis, MO) according to the manufacturer's instructions. Briefly, MTT (5 mg/mL) was added and plates were incubated at 37°C for 4 hr before 100 μL dimethyl sulfoxide (DMSO) was added to each well. Finally, the absorbance of each well was read at a wavelength of 540 nm using a microplate reader.

### Evaluation of apoptotic cell death

For in situ detection of apoptotic cells, TUNEL assay was performed by using the in situ Cell Death Detection Kit (Roche Diagnostics GmbH, Mannheim, Germany) according to the manufacturer's instructions. Cervical cancer cells (1 × 10^4^ cells/well) on 8-chamber slides, and the cells were treated with BV. The cells and tumor tissues were washed with PBS and fixed by incubation in 4% paraformaldehyde in PBS for 1 h at room temperature. Membrane was permeabilized by exposure to 0.1% Triton X-100 in PBS for 5 min at room temperature. For DAPI staining, slides were incubated for 15 min at room temperature in the dark with mounting medium for fluorescence containing DAPI (Vector Laboratories, Inc., Burlingame, CA). The cells were then observed through a fluorescence microscope (Leica Microsystems AG, Wetzlar, Germany).

### Western blot analysis

Cultured cells or tumor tissues were washed twice with 1 × PBS, followed by the addition of 1 ml of PBS, and the cells were scraped into a cold Eppendorf tube. Whole cell extracts and tissue proteins were mixed with sodium dodecyl sulfate (SDS) sample buffer and then subjected to 12% SDS-polyacrylamide gel electrophoresis. The resolved proteins were transferred to a polyvinylidenedifluoride (PVDF) membrane (GE Water and Process technologies, Trevose, PA, USA). Blots were blocked for 1 h at room temperature with 5% (w/v) skim milk in Tris-Buffered Saline Tween-20 [TBST: 10 mM Tris (pH 8.0) and 150 mM NaCl solution containing 0.05% Tween-20]. After a short washing in TBST, the membranes were immunoblotted with the following primary antibodies: caspase-3, caspase-9, caspase-8, TNF Receptor 1 and Bcl-2 (1:1000 dilutions; Cell Signaling, Beverly, MA) and p65, p50, p53, DR3, DR6, Fas, TWEAK, TRAIL, TNF Receptor 2, Fas Ligand and (1:2000 dilutions; Santa Cruz Biotechnology, Santa Cruz, CA). The blots were performed using specific antibodies followed by second antibodies and visualization by chemiluminescence (ECL) detection system.

### Transfection of siRNA

Cervical cancer cells (1×10^4^ cells/well) were plated in 96-well plates and transiently transfected with siRNA, using a mixture of siRNA and the WellFect-EX PLUS reagent in OPTI-MEN, according to the manufacturer's specification (WelGENE, Seoul, Korea). The transfected cells were treated with 5 μg/ml BV for 24 h or 2 hr and then used for detecting cell viability, protein expression and NF-κB activation.

### Electrophoretic mobility shift assay

The DNA binding activity of NF-κB was determined using an electrophoretic mobility shift assay (EMSA) performed as according to the manufacturer's recommendations (Promega). In short, Ca Ski and C33A cells were cultured on 100-mm culture dishes. After treatment with BV (1, 2, and 5 μg/ml) for 2 hr, the cells were washed twice with PBS, followed by the addition of 1 ml of phosphate buffered saline (PBS), and the cells were scraped into a cold Eppendorf tube. Nuclear extracts were prepared and processed for EMSA as previously described [24]. The relative densities of the DNA–protein binding bands were scanned by densitometry using MyImage (SLB), and quantified by Labworks 4.0 software (UVP, Inc., Upland, CA).

### Data analysis

The data were analyzed using the GraphPad Prism 4 ver. 4.03 software (Graph-Pad Software, La Jolla, CA). Data are presented as mean ± SD. The differences in all data were assessed by one-way analysis of variance (ANOVA). When the P value in the ANOVA test indicated statistical significance, the differences were assessed by the Dunnett's test. A value of P ≤ 0.05 was considered to be statistically significant.

## SUPPLEMENTARY FIGURE


