# Supplementary material for: Bee venom inhibits growth of human cervical tumors in mice
Source: Oncotarget. 2015 Jan 23;6(9):7280–92. doi: 10.18632/oncotarget.3110 (PMC4466684; doi:10.18632/oncotarget.3110)
Supplement: Supplementary file 1 [file oncotarget-06-7280-s001.pdf]

SUPPLEMENTARY FIGURE

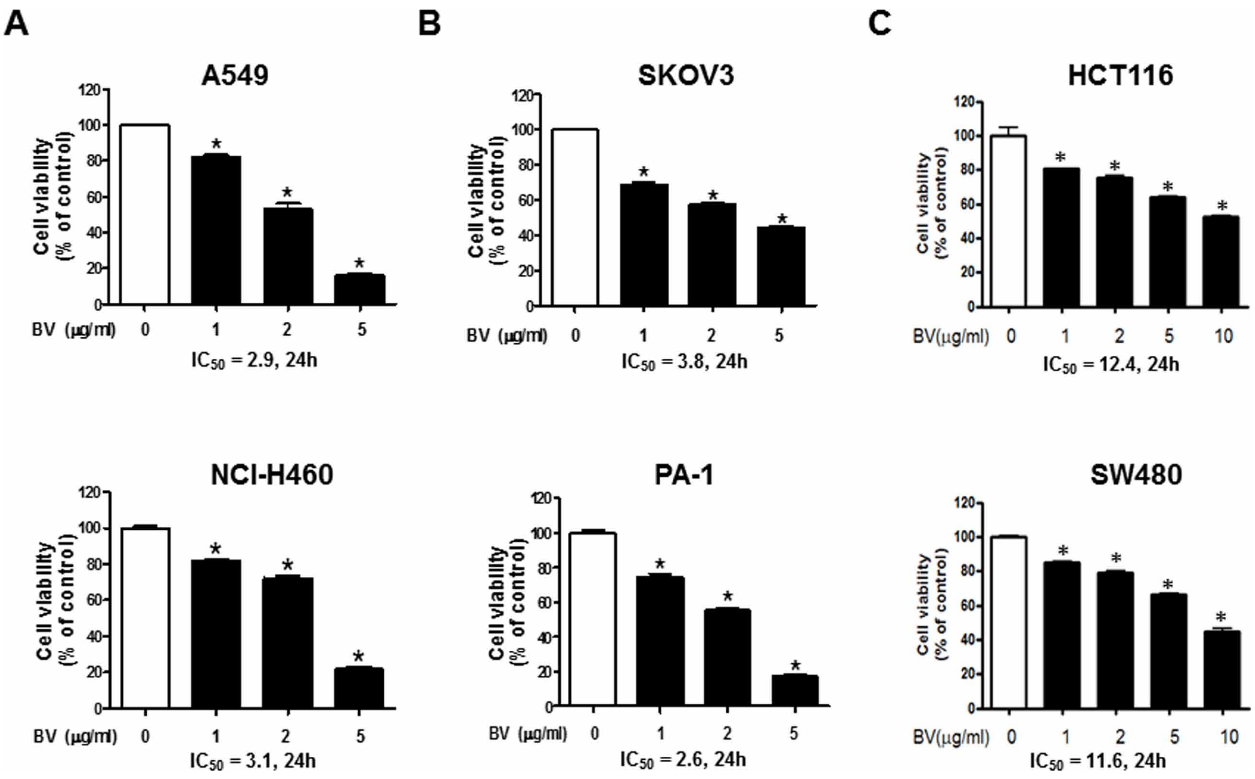

Supplementary Figure S1: Effect of BV on cell viability of various cancer cells. Concentration-dependent effect of BV on the several cancer cells; lung **(A)**, ovarian **(B)** and colon **(C)** after 24 hr treatment. The data were expressed as the mean ± S.D. of three experiments. *($P < 0.05$) indicates statistically significant differences from the control group.
